# Remineralizing effect of NSF on artificial enamel caries

**DOI:** 10.1186/s12903-024-04668-x

**Published:** 2024-08-22

**Authors:** Osama Safwat Mohamed, Mohamed Ashraf Hall, Inas Karawia

**Affiliations:** 1 Dental Prosthesis Manufacture Technology Department, Faculty of Applied Health Sciences Technology, Pharos University, Alexandria, Egypt; 2 Alexandria Dental Research Center, Ministry of Health and Population, Alexandria, Egypt; 3 Pediatric and Community Dentistry Department, Faculty of Dentistry, Pharos University, Alexandria, Egypt

**Keywords:** Dental caries, Remineralization, Fluoride

## Abstract

**Introduction:**

Nanotechnology offers new approaches and endless opportunities for remineralizing tooth decay without being toxic or causing allergies. This study aimed to determine the effect of nanosilver fluoride (NSF) on the remineralization potential of enamel caries-like lesions compared to 5% sodium fluoride varnish in permanent teeth.

**Methods:**

Fifteen teeth (molars and premolars) were gathered, cleaned, and polished using a scaler. After sectioning the teeth mesiodistally and removing the roots, the thirty specimens were subjected to a demineralized solution to induce early enamel lesions and then assigned randomly into two equal groups. The test materials were applied, and then all the specimens were subjected to a pH cycling model for 30 days. DIAGNOdent and surface roughness were investigated, and an evaluation of the enamel Ca and P weight% for Ca/P ratio calculation was done using SEM-EDX to analyze the specimens at the end of the study. The data were analyzed using an independent t-test.

**Results:**

The mean values for the DIAGNOdent measurements for NSF and NaF at baseline and after demineralization were not significantly different (*p* > 0.05). After treatment, NaF varnish showed a significantly higher mean DIAGNOdent measurement (11.8 ± 5.80) than NSF (4.7 ± 1.6). The mean surface roughness of the NaF group (1.64 ± 0.39) was much higher than NSF’s mean surface roughness (1.07 ± 0.21). Specimens treated with NSF had statistically significant smoother surfaces (*p* < 0.001). The NSF group had a higher mean Ca/P ratio (2.9 ± 0.35) than NaF (2.2 ± 0.11). This difference was statistically significant (*p* = 0.012).

**Conclusion:**

The study reveals that nano silver fluoride is a more effective treatment than sodium fluoride varnish in enhancing teeth’s clinical characteristics, particularly in terms of mineral content and surface roughness, suggesting it could be an improved strategy to prevent dental caries and maintain enamel integrity.

## Introduction

Despite advancements in dental treatment, dental caries remains a serious health concern [1]. While the prevalence of dental caries has decreased over the past 25 years, recent studies report an increase in both permanent as well as primary dental caries globally [2, 3]. The ongoing, alternating processes of demineralization and remineralization can result in dental caries [4].

Remineralization may take place in an appropriate state, and the process can be regulated by the degree of apatite mineral saturation of dental plaque and saliva [5]. The mainstay of minimum intervention dentistry (MID) treatment for caries management is the remineralization procedure, which has the benefit of being non-invasive [6]. Such intervention offers to treat lesions that exist within the enamel, furthermore, original enamel defects are unable to heal naturally once they develop into cavities [7].

The administration of topical fluoride is the clinically advised non-invasive caries control strategy for this caries category [6]. Applying topical fluoride is advised to avoid dental cavities [8, 9]. The advancement of early enamel caries lesions, which may be seen as brown or white spots, cannot be effectively halted by topical fluoride [10].

A thorough evaluation found that 5% Sodium Fluoride (NaF) varnish was more efficient in remineralizing early enamel caries. Despite the widespread use of NaF as a remineralizing agent [11, 12], it has some disadvantages such as a burning sensation in the gingiva when fluoride comes in contact with gingival tissue. Furthermore, there is a risk of contact allergy to the patients and the dentist. So, it may be beneficial to explore possible alternatives [13, 14].

Nanotechnology is widely incorporated in several cosmeceutical products like lip creams, whitening creams and anti-aging products. Nanoparticles are considered a zero-dimensional material, and their efficacy comes from their physical and chemical properties [15]. The nanotechnology field introduces a great improvement in dentistry by emerging biocompatible materials that reduce toxicity [16].

Nano-silver fluoride (NSF), which contains fluoride and chitosan, stops and arrests carious lesions without being toxic or causing allergy. Clinical testing on nano-silver fluoride has previously shown that it is efficient in stopping cavities in the enamel and dentin without discoloring the teeth [17]. NSF treatment is inexpensive, easy to administer, and non-invasive. This novel chemical is perfect for application in communities with limited resources in developing countries since it can help improve dental care accessibility without stigmatizing people by making their teeth appear discolored, unlike Silver diamine fluoride (SDF) [18].

Nanotechnology may offer novel strategies and limitless opportunities for the remineralization of submicrometric-sized tooth decay [19]. The preventative and antibacterial effects of nano-silver particles and fluoride have been combined in an experimental formula [20]. Silver nanoparticles (AgNPs), fluoride, and chitosan are present in the reddish-yellow solution that is available for purchase. The main pathogens that promote cariogenic conditions are lactobacilli and mutans streptococci, which have been demonstrated in numerous investigations to be effectively inhibited by chitosan and AgNPs [21]. Fluoride greatly inhibits the production of extracellular polysaccharides by bacteria and obstructs their ability to produce enzymes [22]. The synergy between the constituents of NSF’s formulation explained the success of its caries arrest [23]. It is stated to be eco-friendly, inexpensive, safe, stable for three years, simple to use, and able to be used once a year. Also, this material is easy to utilize, non-invasive, and less technique-sensitive, which makes it a wonderful fit for public health initiatives [24].

Besides the antibacterial effect of NSF, its fluoride component promotes enamel remineralization and prevents the dissolution of its minerals [25]. Also, the presence of silver nanoparticles penetrate the demineralized enamel and increase its hardness to prevent the undesirable acid effect on the enamel [26]. Chitosan, one of NSF ingredients interferes with enamel demineralization [1].

After an in-depth review of the literature, we found that few studies compared NSF with NaF varnish and the results of these studies were conflicting, Akyildiz et al. [27] found that NSF was less effective than sodium fluoride varnish and silver diamine fluoride in remineralizing artificial enamel caries lesions, while El-Desouky et al. concluded that both varnish types showed equal effect in preventing enamel demineralization from acidic challenges [28]. However, a clinical trial in 2023 concluded that NSF varnish decreased caries activity, and DIAGNOdent scores in permanent teeth [29]. Similarly, Deulkar et al. [12] found that NSF varnish, particularly the acidulated form, was more effective than sodium fluoride varnish in remineralizing enamel caries in primary teeth. Furthermore, the effect of NSF was not thoroughly researched without bacteria. Therefore, the current in vitro investigation sought to determine the effect of NSF on the remineralization potential of enamel caries-like lesions compared to 5% NaF varnish in permanent teeth. Our null hypothesis is that there is no difference between NSF and NaF varnish in the remineralizing capacity after 30 days.

## Materials and methods

The Ethics Committee at Pharos University approved this comparative in vitro study under the number (PUA-UREAC-04-3-202). Using G power software, the minimum required sample size was determined to be 28 and was rounded to 30 specimens, with an effect size of 1.3 which was obtained from a previous study that evaluated the prevention capacity of nano-silver fluoride versus sodium fluoride varnish on enamel caries-like lesions [28], alpha error (0.5), and 95% confidence interval.

Sound molars and premolars extracted from humans were gathered from the dental clinics of Pharos University. Teeth with dental cavities, fractures, stains, or cracks were excluded. To ensure the included teeth fulfilled the inclusion criteria, they were meticulously cleaned of blood and debris before being examined. DIAGNOdent was used to examine the specimens’ smooth surface, the laser probe scanned the enamel with pendulum movement without pressure, and the maximum value was recorded (value from 0:14), which indicates a sound tooth, more than 14 indicates the presence of caries [30]. After that, they were kept at room temperature in 2% formaldehyde until needed for use.

### Nano-silver fluoride preparation

Using a magnetic stirrer, chitosan (28.7 ml, 2.5 mg/ml) was melted in an acetic acid solution (1%). Then, it was transferred to an ice bath after filtration, then silver nitrate (1 ml, 0.11 mol/L) was added to the prepared chitosan mixture, and next, sodium borohydride (0.3 ml, 0.8 mol/L) was dropped. The color of the solution changed to yellowish and then reddish brown, indicating the production of AgNPs. The flask was then removed from the ice bath and the fluoride (10,147 ppm fluoride) was added for better stability of the new product [31] .

**Fig. 1:**
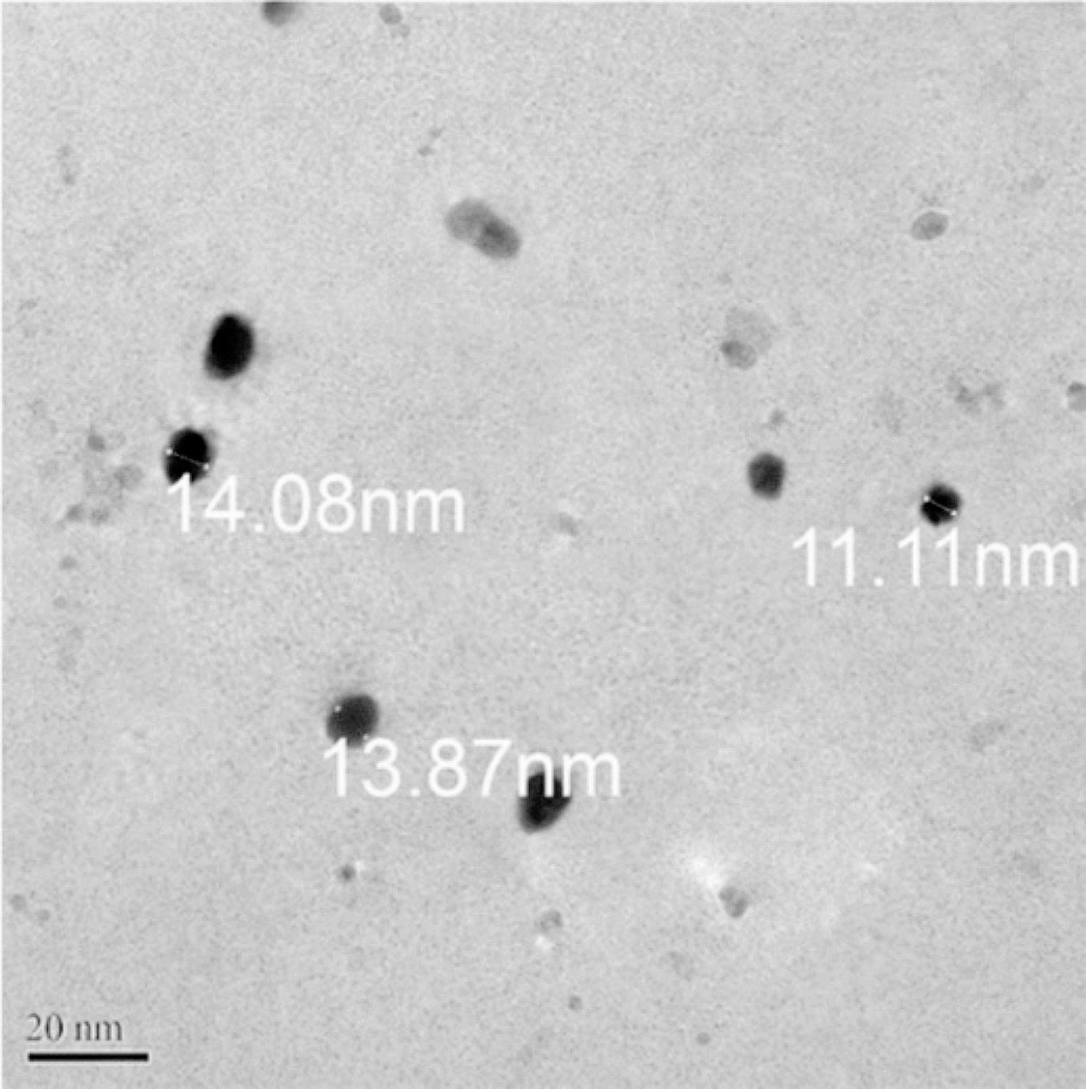
Transmission electron microscopy of the produced specimen reveals the form and size of silver nanoparticles

The size and morphology of AgNPs were determined using a field emission transmission electron microscope (TEM) (JEOL JEM-2100 F). Most of the particles had a spherical form, and most identified particles varied in size from 11 to 14 nm (Fig. 1).

### Specimens preparation

Fifteen teeth were sectioned mesiodistally the roots were removed, and a 4 × 4 mm piece of self-adhesive tape was placed in the center of the middle third of each buccal or lingual surface of each specimen. The surfaces of the specimens were all covered with acid-resistant nail polish. Once the teeth had dried, the self-adhesive tapes were taken off, exposing only a 4 × 4 mm section of enamel on each specimen.

The specimens were demineralized with a demineralizing solution. The demineralizing solution (2.2 mM Ca(NO3)2.2H2O, 50 mM acetic acid and 2.2 mM KH2PO4) [32]. The pH of the solution was adjusted to 4.2 using small amounts of NaOH, pH was recorded daily with a pH meter, and pH was maintained between 4.2 and 4.25 by adding refreshing amounts of HCL 30% solution. The teeth were submerged in a demineralizing solution for three days, and the solution was refreshed daily. The specimens were then analyzed again using DIAGNOdent to confirm demineralization of all the specimens, with results between 13 and 21.

### Grouping and treatment

A randomization list was created to assign each half specimen of the same tooth to be in one of the two groups based on the used treatment.

#### Group 1

Nano-silver fluoride varnish (15 specimens).

#### Group 2

Na fluoride varnish (15 specimens).

Both varnishes were applied to the enamel surface using a small microbrush with a head size of 2 mm for applying a thin layer of varnish on the enamel surface.

Directly following treatment, the specimens were exposed to a pH cycle using demineralizing for 2 h followed by remineralizing solutions (1.5 mM CaCl2, 0.9 mM NaH2PO4, 0.15 M KCL had a pH of 7.0, 1.1 ml/mm2) [32] for 22 h daily till the end of the study after one month.

### Evaluation

DIAGNOdent was used to analyze the specimens again at the end of the study to evaluate the remineralization. While surface roughness was investigated using a surface roughness tester (Surftest 401; Mitutoyo, Kawasaki, Japan), the device has a 5 μm pointer with a 90˚ angle directed to the enamel surface, at a 0.5µ/s constant speed and 4µN measuring force. For each specimen, 3 readings were taken, and the mean values were recorded. Surface roughness evaluation was done at the Faculty of Dentistry, Alexandria University. Finally, the enamel Ca and P weight% assessment for Ca/P ratio calculation was done using Scanning electron microscopy with energy dispersive X-ray spectroscopy (SEM-EDX) (JEOL JSM-IT200 Scanning Electron Microscope- USA) with EDX detector optimized at working distance 10 mm, at Alexandria University’s Faculty of Science.

### Statistical analysis

Data were analyzed with IBM SPSS for Windows version 25.0 (IBM Corp., Armonk, New York, USA). The Kolmogorov-Smirnov test was used to determine normality, and data were presented as mean ± SD. The differences between the test groups were examined using an independent t-test. In all statistical analysis, with a p-value of 0.05 or below, the results were considered statistically significant.

## Results

The clinical characteristics of Groups 1 and 2 are compared in Table 1 with the associated p-values and the outcomes of the t-tests. Groups 1 and 2 showed comparable mean values for the DIAGNOdent baseline measurements (Group 1: 6.13 ± 1.9; Group 2: 6.13 ± 1.5), and a t-test results in a non-significant result (t = 0.0, *p* = 1). Group 1 displayed a mean DIAGNOdent measurement of 16.6 ± 2.7 after demineralization, whereas Group 2 displayed a mean of 15.5 ± 2.5, the difference (t = 1.12, *p* = 0.27) was not statistically significant.

After treatment, nevertheless, there were noticeable variations between the groups. Group 2’s mean DIAGNOdent measurement of 11.8 ± 5.8 (t = 16.04, *p* < 0.001) was substantially higher than Group 1’s mean of 4.7 ± 1.6. Regarding surface roughness, Group 2’s mean (1.64 ± 0.39) was much higher than Group 1’s mean (1.07 ± 0.21). It appeared that Group 1 has smoother surfaces, and this difference was statistically significant (t = -5.008, *p* < 0.001).

Group 1 had a greater mean value of 44.3 ± 2.4 for Ca weight% than Group 2 (36.9 ± 1.87). Higher calcium levels were found in Group 1, as indicated by this statistically significant difference (t = 9.28, *p* < 0.001). Group 1 showed a lower mean value of 15.6 ± 1.2 for P weight% than Group 2 (16.9 ± 0.37). In comparison to Group 2, it appeared to have been a statistically significant decrease in phosphorus level in Group 1 (t = -4.15, *p* < 0.001). Group 1 had a higher mean Ca/P ratio (2.9 ± 0.35) than Group 2 (2.2 ± 0.11), this difference was statistically significant (t = 7.27, *p* = 0.012).

Overall, the findings demonstrate significant differences between the groups across various clinical characteristics, following treatment and concerning surface roughness, calcium, and phosphorus levels.

**Table 1:** Comparison between the study groups

Clinical characteristics	Group 1	Group 2	t-test	*p*-value
	Mean (SD)	Mean (SD)		
DIAGNOdent baseline	6.13(1.9)	6.13(1.5)	0.0	1
DIAGNOdent After demineralization	16.6(2.7)	15.5(2.5)	1.12	0.27
DIAGNOdent after treatment	4.7(1.6)	11.8(5.8)	16.04	< 0.001*
surface roughness	1.07(0.21)	1.64(0.39)	-5.008	< 0.001*
Ca weight%	44.3(2.4)	36.9(1.87)	9.28	< 0.001*
P weight%	15.6(1.2)	16.9(0.37)	-4.15	< 0.001*
Ca/P	2.9(0.35)	2.2(0.11)	7.27	0.012*

Group 1: Nano-silver fluoride varnish

Group 2: Na fluoride varnish

*: Significance at p-value ≤ 0.05

## Discussion

Since dental caries is common and negatively affects the quality of life, it presents a challenge [33]. It represents a global public health problem with a global high prevalence rate of 46.2% and 53.8% in 2020 in primary and permanent teeth, respectively [2]. As a result, appropriate procedures should be adopted to manage at all levels and to ameliorate the condition. Finding effective treatment and preventative methods is essential to achieving the goal of reducing dental caries [34]. The most commonly used topical fluoride agent is sodium fluoride (NaF) varnish, which has 22,600 ppm fluoride [35]. When applied, NaF varnish combines with hydroxyapatite crystals to form a layer of calcium fluoride that covers the HAP lattice. This event, known as choking off, allows for a continuous release of fluoride [36].

In the current study, NSF was prepared with a concentration of 10,147 ppm fluoride, as it was reported that 10,000 ppm or above has an acceptable remineralization effect [37]. NSF is a novel anti-caries agent that has emerged as a consequence of the application of nanoscience and technology in dentistry [24]. The main microorganisms linked to the development of dental caries, Mutans streptococci and Lactobacilli, are effectively inhibited by this new anti-caries substance, which is safe for human usage [18] .

The purpose of this work was to compare the in vitro remineralization capacity of NSF on enamel caries-like lesions to that of 5% NaF in permanent teeth. Based on significant differences between the groups in a number of clinical variables, including DIAGNOdent reading, surface roughness and calcium and phosphorus enamel levels. After treatment, the results showed that NSF varnish is more effective than NaF varnish, so the null hypothesis was rejected.

The results of the current study found NSF had a significant remineralization potential than NaF varnish in the treatment of artificially demineralized enamel in the three outcomes: DIAGNOdent reading values, Surface roughness and Ca/P ratio. This is consistent with previously published clinical experiments [29, 38]. A previous systematic review found that, over 12 months, demonstrated a significantly higher degree of improvement in diagnoses in NSF compared to NaF [39].

However, Akyildiz et al. [27] found that NaF had a better effect than NSF in the remineralization effect on enamel after 7 days, the inconsistent results with the current study may be due to different pH cycling protocols, measured outcome and follow-up periods. In the current study, both (NSF and NaF) showed the remineralizing effect of the enamel specimens according to the DIAGNOdent reading but the effect of NSF was better after one month which may reflect the prolonged effect of NSF than NaF varnish. NaF varnish is recommended to be applied at least every 6 months, and it is preferred to be 3 to 4 month intervals [40], while NSF may be recommended to be used once a year [41]. Teixeira et al. [21] found no statistically significant difference in enamel demineralization prevention between NSF and NaF dentifrices, despite the fact that NaF had a smaller percentage of microhardness variation. The utilization of distinct pH cycling protocols and the frequent application of dentifrice slurries prior to each pH cycle may account for this. In addition, it was found that NSF and NaF varnish had comparable effects in preventing enamel demineralization brought on by the artificial cariogenic challenge, according to El-Desouky et al.‘s study [28]. This is largely explained by how the high fluoride concentration of both treatment materials affected the surface of the specimens. Furthermore, the application of the materials resulted in the creation of a layer resembling calcium fluoride (CaF2), which served as a reservoir throughout the demineralization phases and gradually released fluoride to shield the enamel surface from erosion. Fluoride drew calcium and phosphate ions from the remineralizing fluid during the remineralization phases, generating a heavily mineralized layer on the surface of the caries-like lesion [42].

Loss of tooth minerals leads to enamel surface roughness that is assigned to the hydroxyapatite crystals dissolution during the demineralization, which establishes microporosities in the enamel superficial layer. When sufficient Ca and P ions are presented in saliva, remineralization occurs and repairs the dissolved hydroxyapatite structure [43]. Minimally invasive treatments are used to impact the rate of demineralization and remineralization. When remineralization exceeds the rate of demineralization, results in a smoother surface [44]. This may be the cause of better surface roughness results for the NSF group in the current study.

The small nanoparticles of NSF facilitate the penetration of fluoride into the enamel [37]. This means that after applying NSF, calcium fluoride should be expected. The outcomes of a related investigation [12] demonstrated the effectiveness of NSF and 5% NaF in helping primary teeth’s enamel caries remineralization. In the 5% NaF group with no silver content, an energy dispersive X-ray spectrophotometry (EDX) examination found a fluoride concentration of 5.45 weight%, while there was 14.64 weight% of fluoride in the NSF group. The fluoride and silver levels on the tooth’s surface validate the mineral deposition there, which starts the remineralization process [5]. Additionally, the presence of chitosan as one of the ingredients of NSF controls the process of biomineralization by the interaction with minerals producing organized crystallization [45].

## Conclusion

Based on the results of this study, nano silver fluoride seems to be a more effective therapy than sodium fluoride varnish in terms of enhancing the clinical characteristics of teeth, especially with regard to mineral content and surface roughness. This implies that the use of nano silver fluoride may be an improved strategy to prevent dental caries and maintain the integrity of enamel. Before making clear therapeutic recommendations, it is important to take into account the larger context of these findings, which includes the sample size, the length of the trial, and any possible side effects or long-term consequences.

### Recommendation

With the limitation of this in vitro study, more studies are needed to evaluate the effect of NSF on enamel by measuring fluoride and silver content. Also, for the limitations of EDS, studies using transverse microradiography or micro-computed tomography assessment should be conducted. Studies conducted in the clinical site are recommended to ensure the effectiveness of NSF in comparison with other remineralizing materials.

## Data Availability

Availability of data and materialsThe datasets used and/or analyzed during the current study are available from the corresponding author on reasonable request.
